# A Terpene Synthase Is Involved in the Synthesis of the Volatile Organic Compound Sodorifen of *Serratia plymuthica* 4Rx13

**DOI:** 10.3389/fmicb.2016.00737

**Published:** 2016-05-19

**Authors:** Dajana Domik, Andrea Thürmer, Teresa Weise, Wolfgang Brandt, Rolf Daniel, Birgit Piechulla

**Affiliations:** ^1^Institute for Biological Sciences, University of RostockRostock, Germany; ^2^Institute of Microbiology and Genetics, Applied Microbiology and Göttingen Genomics Laboratory, University of GöttingenGöttingen, Germany; ^3^Euroimmun AGLübeck, Germany; ^4^Leibniz Institute of Plant BiochemistryHalle, Germany

**Keywords:** *Serratia*, bacterial terpene cyclase, sodorifen, volatile organic compound

## Abstract

Bacteria release a plethora of volatile organic compounds, including compounds with extraordinary structures. Sodorifen (IUPAC name: 1,2,4,5,6,7,8-heptamethyl-3-methylenebicyclo[3.2.1]oct-6-ene) is a recently identified and unusual volatile hydrocarbon that is emitted by the rhizobacterium *Serratia plymuthica* 4R×13. Sodorifen comprises a bicyclic ring structure solely consisting of carbon and hydrogen atoms, where every carbon atom of the skeleton is substituted with either a methyl or a methylene group. This unusual feature of sodorifen made a prediction of its biosynthetic origin very difficult and so far its biosynthesis is unknown. To unravel the biosynthetic pathway we performed genome and transcriptome analyses to identify candidate genes. One knockout mutant (*SOD_c20750*) showed the desired negative sodorifen phenotype. Here it was shown for the first time that this gene is indispensable for the synthesis of sodorifen and strongly supports the hypothesis that sodorifen descends from the terpene metabolism. *SOD_c20750* is the first bacterial terpene cyclase isolated from *Serratia* spp. and Enterobacteriales. Homology modeling revealed a 3D structure, which exhibits a functional role of amino acids for intermediate cation stabilization (W325) and putative proton acception (Y332). Moreover, the size and hydrophobicity of the active site strongly indicates that indeed the enzyme may catalyze the unusual compound sodorifen.

## Introduction

Plant and animal volatile emissions and volatile–based interactions are well known. Only recently it emerged that microorganisms also emanate a wealth of diverse volatiles and complex volatile mixtures (Effmert et al., 2012). Volatile organic compounds (VOCs) produced in the microbial world occur in the biosphere in concentrations over several orders of magnitude, and they are ideal infochemicals because their spheres of actions include ‘aqueous’ as well as ‘atmospheric’ diffusion (Wheatley, 2002). The ecological functions of microbial VOCs (mVOCs) are largely unknown, but in principal they can act as signals for communication between various organisms, or serve as attractant or defense compounds. For example, VOCs of rhizobacteria inhibit the growth of various fungi by reducing sporulation, inhibiting mycelium growth, altering hyphal morphology, or inhibiting spore germination (Alström, 2001; Wheatley, 2002; Fernando et al., 2005; Kai et al., 2007, 2009). Microbial VOCs also affect morphology and physiology of *Arabidopsis thaliana* (Wenke et al., 2012).

**FIGURE 1:**
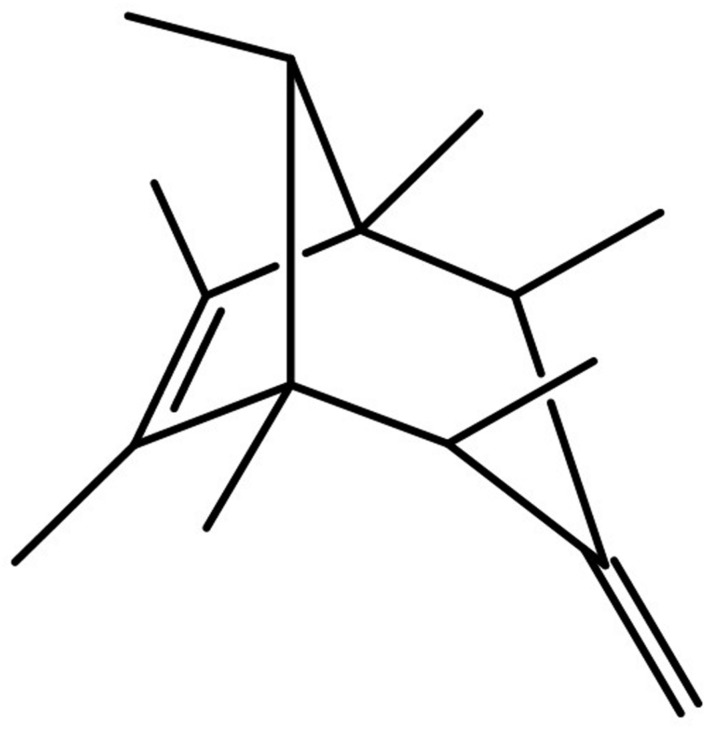
**Structure of sodorifen which is emitted by *Serratia plymuthica* 4Rx13 (von Reuss et al., 2010)**.

Analysis of the production and emission of blends from rhizobacterial and plant-colonizing bacterial isolates revealed VOC mixtures of different complexity, e.g., *Serratia plymuthica* 4Rx13 emits at least 100 compounds (Kai et al., 2010). A literature survey revealed the first microbial volatile database ‘mVOC’ (Lemfack et al., 2014) comprising to date approximately 1500 mVOCs released from ca. 450 bacterial and fungal species/isolates. Many more mVOCs are expected to be synthesized in nature, because (i) more than 10^17^ bacterial species exist on earth, and (ii) complexity and diversity of volatile blends depends on the metabolic activities, e.g., correlating with growth conditions, of the organisms. Interestingly, many unidentified compounds are emitted by bacteria, which remain to be structurally elucidated. In this regard, our present interest was focused on *S. plymuthica* 4Rx13, since its VOC profile was dominated by one single new compound (relative contribution ca. 45%) (Kai et al., 2010). While the biological and ecological function of this compound remains elusive, we used a combination of MS and NMR techniques to elucidate the structure of the target compound: the volatile is 1,2,4,5,6,7,8-heptamethyl-3-methylenebicyclo[3.2.1]oct-6-ene (sodorifen) which was confirmed by total synthesis (von Reuss et al., 2010). Sodorifen has an extremely unusual structure, it is a polymethylated bicyclus (**Figure 1**) and a prediction of its biosynthesis was previously impossible. Consequently it was the goal to unravel the underlying biosynthetic pathway(s).

## Materials and Methods

### Bacterial Strains, Media, and Growth Conditions

The investigations were conducted with the following species: *Serratia plymuthica* 4Rx13 and HRO-C48 were isolated from the rhizosphere of *Brassica napus* and *S. plymuthica* 3Re-4-18 was isolated from the endorhiza of *Solanum tuberosum* (Prof. Gabriele Berg, Institute of Environmental Biotechnology, University of Graz, Austria); *S. plymuthica* V4 was isolated from a biofilm of pasteurizer plates of milk processing machine (provided by R. Kolter, Boston, Harvard Medical School, USA; Cleto et al., 2014); and *S. plymuthica* AS9 and 12 were isolated from rapeseed roots grown in Sweden (provided by Sadhna Alström; Neupane et al., 2012). All bacteria were cultivated at 30°C in a complex medium (NB II; Carl Roth, Karlsruhe, Germany; 3.5 g/l peptone from casein, 2.5 g/l peptone from meat, 2.5 g/l peptone from gelatin, 1.5 g/l yeast extract, 5 g/l NaCl, 15 g/l agar-agar, pH 7.2) or in liquid nutrient broth (NB II without agar-agar). The OD_600_ was determined with a photometer (UV/Visible Spectrometer, Ultrospec 2000; Pharmacia Biotech), and the number of colony forming units (CFUs) was calculated by plating dilution series.

### Volatile Collection

Bacteria were cultivated in 6 ml NB II overnight at 30°C and 170 rpm, and once OD_600_ = 0.5–1 was reached transferred into a 100 ml modified Erlenmeyer flask of the VOC-collection system (Kai et al., 2010). Volatile compounds of the headspace of three 24 h intervals (air flow 1.1 ml/min) were trapped on the absorbent SuperQ (50 mg, brand new SuperQ to avoid cross-contamination; Alltech, IL, USA). The trapped VOCs were eluted with dichloromethane (Carl Roth, Karlsruhe, Germany). Nonyl acetate (Carl Roth, Karlsruhe, Germany) was added as internal standard (stock solution 5 ng/10 μg), peak in the chromatogram corresponds to 5 ng.

### Analysis of Volatile Compounds by Gas Chromatography Mass Spectrometry

Samples were analyzed with the Shimadzu GC/MS-QP500 system (Kyoto, Japan), equipped with a DB5-MS column (60 m × 0.25 mm × 0.25 μm; J&W Scientific, Folsom, CA, USA). Before sample analysis always a control run was performed to preclude detection of residual compounds of previous analyses. Samples of 1 μl were injected splitless or with a split ratio of 1:25 at 200°C with a solvent delay of 2 min using a CTC autosampler (CTC Analytics, Zwingen, Switzerland). Helium was used as a carrier gas at a flow rate of 1.1 ml/min. The capillary was equipped with diphenyl (dimethyl) polysiloxan with 5% phenyl groups (DB05). The mass fragments were selected based on their mass to charge ratio using a quadrupole mass analyzer. Mass spectra were recorded using scan mode (70 eV, *m/z* = 40–280). The selected masses were analyzed with the software Lab Solution (Shimadzu, Duisburg, Germany). To determine the structure of the volatile compounds, the mass spectra were compared with those of the Mass Spectral Library of the National Institute of Standards and Technology (NIST107).

### Polymerase Chain Reaction

The amplification of DNA fragments was conducted by polymerase chain reaction (PCR). A standard PCR was performed using Taq polymerase I (overexpressed; Department of Biochemistry, University of Rostock, Germany). DNA was isolated with NucleoSpin Tissue Kit (Macherey-Nagel, Düren, Germany). For long amplification products (>3 kb), the High-Fidelity Polymerase *Pfu* or Phusion^®^ (Fermentas, St. Leon-Rot, Germany) was used. Amplification reactions were conducted in a total volume of 50 μl, consisting of 1 μl of 10 mM dNTP-Mix, 1 μl of each primer, 1 μl template DNA, 0.5 μl polymerase with or without the proofreading function, 10 μl buffer, and 35 μl nuclease-free water. Primers were designed according to the gene properties (temperature, GC content, and secondary structures) and were usually 21 nucleotides long, with the exception of the oligonucleotides for mutagenesis, which were 72 nucleotides long. A complete list of primers can be found in **Supplementary Table S3**. The annealing temperature for the amplification of DNA depended on the temperature of the primers (x), and the elongation time (y) was selected according to the size of the gene or DNA (1 kb/min). All primers were ordered from Sigma–Aldrich (St. Louis, MO, USA) or Invitrogen (Carlsbad, CA, USA). The PCR was performed in a Biometra PCR-cycler (Göttingen, Germany) or a Hybaid PCR-cycler (Ulm, Germany). Buffer, MgCl_2_, dNTPs, and polymerase were obtained from Thermo Fisher Scientific (Waltham, MA, USA). The PCR program for the Taq polymerase was performed for 30 cycles (94°C for 2 min, 94°C for 30 s, x for y, 72°C for 1 min and 72°C for 10 min). The PCR program for the Phusion^®^ polymerase was performed for 35 cycles (98°C for 30 s, 98°C for 10 s, x for 30 s, 72°C for 90 s, 72°C for 10 min).

### Mutagenesis

Mutagenesis was performed using the manufacturer’s protocol of the “Quick and Easy Gene Deletion Kit” (Gene Bridges, Heidelberg, Germany). An antibiotic cassette was introduced via homologous recombination, which was established for *S. plymuthica* 4Rx13 in our lab. The method depends on a Red/ET system (Zhang et al., 2000) where two helper plasmids (pRed/ET and pFRT) were used for generating single mutants, and an additional plasmid (Flpe-708) was used to produce double mutants. For the gene disruption, primers of 71–73 nucleotides were used (Supplementary Table S3). They consisted of 50 nucleotides homologous to the gene and 21–23 nucleotides homologous to the helper plasmid pFRT, which contained a resistance cassette. The 50 nucleotides flanked the FRT-PGK-gb2-neo-FRT cassette at each site of the kanamycin resistance. This 1700 bp DNA construct was obtained by high fidelity PCR, eluted from the gel (NucleoSpin Gel and PCR Clean-up; Macherey-Nagel, Düren, Germany), and then integrated into the target gene. The helper plasmid pRed/ET was required to integrate the functional cassette by homologous recombination. The plasmid DNA was introduced via electroporation into competent cells of *S. plymuthica* 4Rx13. The mutants were verified by using specific primer combinations that showed the presence of the functional cassette (FRT primer) as well the exact integration site (FRT primer and gene-specific primer) in the gene.

The electroporation: competent cells were mixed with 1 to 2 μl DNA incubated for 20 min on ice together with a control (without DNA) and transferred into sterile cuvettes (electrode distance 0.2 cm, Peqlab, Erlangen, Germany). For introducing the extrinsic DNA (resistance cassette) into competent cells high voltage (pulse control 200 Ω, capacity 25 μF and voltage of 2500 V) was applied with the Gene Pulser II (Bio Rad, Munich, Germany) (Dower et al., 1988). Immediately after the pulse pre-warmed SOC-Medium (0.5% yeast extract, 2% tryptone, 10 mM NaCl, 2.5 mM KCl +1 M MgCl_2_, 1 M MgSO_4_ and 1 M glucose, Carl Roth GmbH, Karlsruhe, Germany) was added and solution was transferred into test tubes (incubation at 37°C for 4 h, at 170 rpm). Cells were harvested (11.000 g, 1 min) and aliquots of the mixture were plated onto solid medium supplemented with kanamycin (50 μg/ml, Roth, Karlsruhe, Germany). Plates were incubated for at least 2 days at 37°C and colonies were analyzed by PCR for the introduced resistance cassette. Subsequently the VOC profile was monitored.

### Plasmid-Assisted Complementation of Mutants

The gene function of the mutants was restored through the introduction of a plasmid containing the wild type gene. This wild type gene was amplified by PCR (Phusion^®^ Polymerase; Thermo Fisher Scientific, Waltham, MA, USA), restriction sites of *Eco*RI and *Bam*HI were added, and subcloned into the vector pJET (CloneJET PCR Cloning Kit; Thermo Fisher Scientific). The modified pJET plasmid was introduced into chemically competent cells of *Escherichia coli* XL-1 Blue. The presence of modified pJET was verified in cells grown on agar supplemented with ampicillin (100 μg/ml). Using gene-specific primers a PCR product was obtained and sequenced (GATC Biotech, Konstanz, Germany). The modified pJET and the vector pUC19 were digested with *Eco*RI and *Bam*HI (Thermo Fisher Scientific, Waltham, MA, USA), and the gene fragment from pJET was ligated into the pUC19 vector (own stock; Department of Biochemistry, University of Rostock, Germany). Different promoters were used: (i) *E. coli lac* promoter of the plasmid pUC19, (ii) *S. plymuthica* 4Rx13 gapDH promoter corresponding to 500 bp upstream of the *gapdh* gene (in frame), and (iii) *S. plymuthica* 4Rx13 promoter corresponding to 500 bp upstream of the gene *SOD_c20780* (in frame). The modified pUC19 vector was inserted into electrocompetent cells of the mutant, and positive clones were selected on ampicillin agar plates. The correct insertion of the construct (gene + promoter) was checked by PCR. Afterward the VOC profiles of two independent clones were monitored over 72 h.

### RNA Isolation

Colonies of the isolates 4Rx13 and AS9 were inoculated for 24 h in 6 ml minimal medium supplemented with 55 mM succinate (Carl Roth, Karlsruhe, Germany) at 30°C and 170 rpm. The cells were then introduced into 100 ml fresh minimal medium with a final OD_600_ = 0.005 and incubated. One culture was harvested after 24 h and another after 48 h (5000 × *g* and 4°C for 20 min). The sediment was treated with the NucleoSpin RNA II Kit (Machery-Nagel, Düren, Germany) to isolate total RNA. The RNA was stored at -70°C before it was used for the transcriptome analysis.

### Genome Sequencing

Genome sequencing was conducted by the Göttingen Genomics Laboratory (G2L) (Göttingen, Germany). Shotgun libraries were generated using the Nextera DNA Sample Preparation kit, following the manufacturer’s instructions (Illumina, San Diego, CA, USA). The whole genome of *S. plymuthica* HRO-C48 was sequenced with the Genome Analyzer IIx (Illumina, San Diego, CA, USA). The library was sequenced in a 112 bp paired-end single-indexed run, resulting in 9.5 and 7.6 mio paired-end reads, respectively. All shotgun reads were assembled *de novo* using the software Ray (v1.1.0; Boisvert et al., 2010). The draft genome of *S. plymuthica* HRO-C48 consisted of 62 contigs (>500 bp) with a genome size of 5.4 Mb. The open reading frames (ORFs) were predicted with the software Glimmer (Delcher et al., 1999). Gene annotation was performed automatically by transferring the existing annotations of the reference genome of *S. plymuthica* 4Rx13. To identify putative orthologous genes, bidirectional BLAST analysis was used to compare the encoded protein sequences of seven whole genome protein datasets for the following *Serratia* species: CP007439.1 *S. plymuthica* V4, CP002773.1 *S. plymuthica* AS9, LFJS01000012.1 *S. marcescens* DB11, NC_009832.1 *S. proteomaculans* 568, NZ_GG753567.1 *S. odorifera* DSMZ 4582, NZ_AJB000088.1 *S. plymuthica* PRI-2C, and NC_021591.1 *S. plymuthica* 4Rx13. The contigs of isolate HRO-C48 (accession number LTDN00000000) were arranged according to the existing reference genome of *S. plymuthica* 4Rx13 using Mauve Multiple Genome Alignment Software (Darling et al., 2010).

### Genome Comparison

The BigBag software tool (bidirectional BLAST) was used to distinguish between the core and pan genome (Altschul et al., 1990; Sayers et al., 2009). For visualization and comparison of the genomes of the *Serratia* spp., the program Artemis (Rutherford et al., 2000) and the DNAplotter (Carver et al., 2009) of the Sanger Institute^1^ were used.

### Transcriptome Analysis

A transcriptome sequencing approach was applied to compare differentially expressed genes of a sodorifen-producer (S. plymuthica 4Rx13) and a non-producer (*S. plymuthica* AS9). Sequencing and library construction were carried out by G2L (Göttingen, Germany). Strand-specific cDNA libraries were generated with the NEBNext^®^ Ultra^TM^ Directional RNA Library Prep Kit of Illumina^®^ (New England Biolabs, Ipswich, MA, USA), and including the removal of rRNA by the Ribo-Zero rRNA Removal Kit^®^ (Bacteria, Illumina, San Diego, CA, USA). Libraries were sequenced on a Genome Analyzer IIx (Illumina, San Diego, CA, USA) and shotgun transcriptomic reads were mapped to the reference genome sequence using Bowtie 2 (v1.0.0; Langmead et al., 2009). Mapping results were used to compare differentially expressed genes. Application of the Transcriptome Viewer (TraV) (Dietrich et al., 2014) visualized the data in tables or plots. To compare the RNASeq experiments, the mapped data were normalized and presented as NPKM (nucleotide activities per kilobase of exon model per million mapped reads), which represents the transcriptional activity of the identified regions.

### Homology Modeling

3D-protein structure homology modeling of the *Serratia plymuthica* 4Rx13 terpene cyclase was performed with YASARA (Krieger et al., 2009). A search for templates in the protein database (Berman et al., 2000) revealed one appropriate X-ray template, the selinadiene synthase, which was complexed with dihydrofarnesyl pyrophosphate from *Streptomyces pristinaespiralis* (pdb-code 4OKZ) (Baer et al., 2014). Based on this X-ray structure three homology models of the *S. plymuthica* terpene cyclase were created, two of them were based on slightly alternative sequence alignments including secondary structure predictions, and a final hybrid model joining was extracted from the best folded fragments of both initial models. The latter one was used for final refinements and docking of the product sodorifen into the active site. Since the template structure was co-crystallized with the ligand dihydrofarnesyl pyrophosphate, this information was automatically transferred into the homology model of *S. plymuthica* by YASARA. The ligand, however, was manually modified to farnesyl pyrophosphate using the molecular modeling environment program MOE 2014.09^2^ and was subsequently optimized in the active site pocket using fixed coordinates of the protein. Sodorifen was docked into the active site using the program MOE as well. The quality of the homology model was evaluated using PROCHECK (Laskowski et al., 1993) and ProSA II (Sippl, 1990, 1993). The model is of excellent quality with 91.9% residues being in the most favored region of the Ramachandran plot and no outlier were recognized. The ProSA energy graphs are all in negative range and the calculated z-scores are in the range of natively folded proteins.

## Results

The aim of this research was to shed light on the biosynthesis of the unique and structurally unusual mVOC sodorifen emitted by *Serratia plymuthica* 4Rx13 (formerly *S. odorifera*).

### Sodorifen Emission in Various *Serratia* Species

**FIGURE 2:**
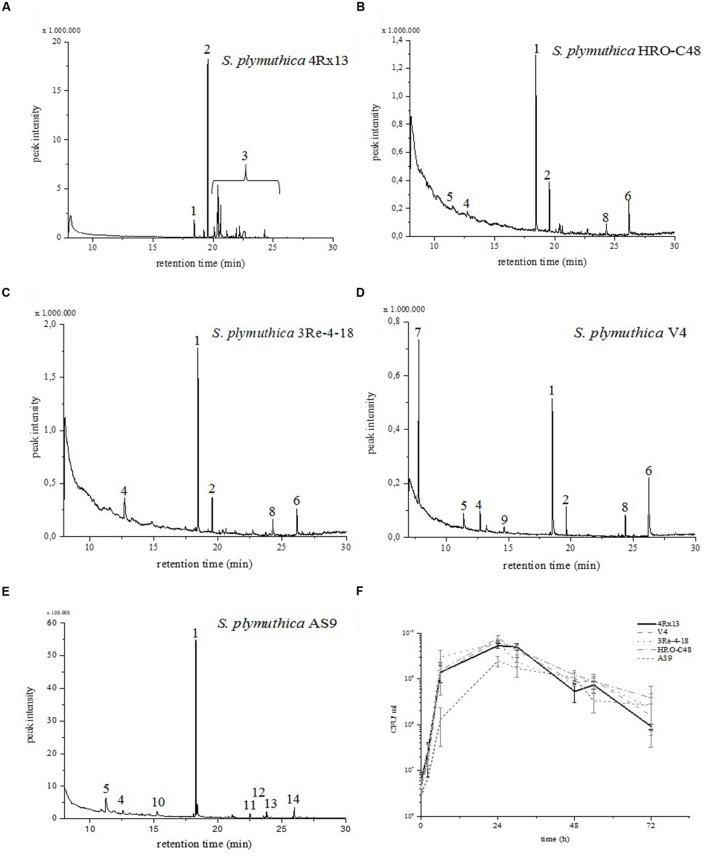
**VOC profiles and growth curves of *Serratia plymuthica* isolates.** The emitted volatiles of the headspaces of **(A)** *S. plymuthica* 4Rx13, **(B)** *S. plymuthica* HRO-C48, **(C)** *S. plymuthica* 3Re-4–18, **(D)** *S. plymuthica* V4, and **(E)** *S. plymuthica* AS9 were harvested in a VOC collection system when strains grew in a complex medium (NB II). Volatiles were analyzed by GCMS (VOC trapping time interval 24–48 h). Compounds were identified by comparing the mass spectra with those filed in the Nist107 library. (#1) nonyl acetate was internal standard (peak corresponds to 5 ng), (#2) sodorifen, (#3) sodorifen isomers, (#4) dimethyl trisulfide, (#5) pyrazine, (#6) hexadecen-1-ol, (#7) dimethyl disulfide, (#8) 2-nonanone, (#9) diethyl ester, (#10) 2-hexanone, (#11) 2-phenylethanol, (#12) undecanone, (#13) 2-butanone, and (#14) n-heptadecyl ester. **(F)** The growth curves of all five *S. plymuthica* isolates were obtained by determining CFU/ml over the time interval 0–72 h. Standard deviations were calculated (*n* = 3).

The genus *Serratia* is known to produce a rich spectrum of VOCs (Kai et al., 2010). Previous investigations of *S. plymuthica* 4Rx13 have illustrated that the VOC profiles of this species is quantitatively and qualitatively different from other *Serratia* spp. under similar growth conditions (Weise et al., 2014). The main compound that is released by *S. plymuthica* 4Rx13 is sodorifen. Here, additional isolates of *S. plymuthica* were screened in order to find other sodorifen producers. The VOC profiles were monitored in three different intervals: 0–24, 24–48, and 48–72 h. The *S. plymuthica* isolates HRO-C48, 3Re- 4–18, and V4 emit sodorifen in different quantities (**Figure 2**, peak #2). *S. plymuthica* 4Rx13 emits sodorifen at very high levels, while *S. plymuthica* 3Re-4-18, HRO-C48 and V4 emit 0.77 – 5.33% of the amount compared to isolate 4Rx13 (**Table 1**). Furthermore, the VOC profile of isolate 4Rx13 exhibits a higher complexity than the other isolates (**Figure 2**). Isolate AS9 lacked sodorifen emission.

**Table 1:** Relative sodorifen emissions in *Serratia plymuthica* isolates.

Time interval (h)	*Serratia plymuthica* isolates				
	4Rx13	HRO-C48	3Re-4–18	V4	AS9
0–24	100%	3.70%	5.33%	0.98%	0%
24–48	100%	2.00%	1.28%	0.77%	0%
48–72	100%	3.07%	3.72%	0.92%	0%


*The sodorifen in the headspace of the isolates HRO-C48, 3Re-4-18, V4, AS9, and 4Rx13 was determined. Bacteria were cultivated in complex medium (NB). The trapped volatiles of three time intervals: 0–24, 24–48, and 48–72 h were eluted and analyzed via GCMS. The relative sodorifen levels were calculated by setting the peak emission of *S. plymuthica* 4Rx13 to 100%.*

It has previously been shown that the compounds dimethyl trisulfide, dimethyl disulfide and pyrazine are produced by 4Rx13 (Weise et al., 2014). This study showed that dimethyl trisulfide (#4) was also found in the profiles of the isolates HRO-C48, 3Re-4–18, V4 and AS9; dimethyl disulfide (#7) was highly emitted by isolate V4, and pyrazine (#5) was released by HRO-C48, V4, and AS9. Furthermore we could show that hexadecen-1-ol (#6) is emitted by isolates 3Re-4-18, V4, and AS9. Isolate AS9 emitted other compounds (2-hexanone (#10), 2-phenylethanol (#11), undecanone (#12), 2- butanone (#13), n-heptadecyl ester (#14), and the general emission is significantly lower than of the other isolates (**Figure 2E**). All sodorifen producers showed similar growth curves, while the non-producer AS9, grew slower in the first 24 h (**Figure 2F**).

### Genome Comparison of *Serratia* Sodorifen Producer and Non-producer Strains

A comparative genome analysis of *S. plymuthica* 4Rx13 with the non-producer isolates *S*. *marcescens* Db11 (LFJS01000012.1), *S. proteamaculans 568 (NC_009832.1)*, *S. odorifera* DSM 4582 (NZ_GG753567.1), *S. plymuthica* PRI-2C (NZ_AJB000088.1), and AS9 (NC_021659.1) was previously carried out by Weise et al. (2014), resulting in the identification of 246 unique ORFs. Among these unique ORFs, 138 genes were annotated as hypothetical proteins, which were of particular interest as they could potentially be involved in the biosynthesis of the structurally unusual compound sodorifen. To further narrow down the number of candidate genes, an additional sodorifen producer isolate, HRO-C48 was sequenced to find common genes with the genome of the isolate 4Rx13 that may be involved in the biosynthesis of sodorifen (**Supplementary Table S1**). Another recently sequenced sodorifen producer, isolate *S. plymuthica* V4 (CP007439.1) was also included into the genome comparison (Cleto et al., 2014). An overview of shared and unique genes was obtained by bidirectional Biblast, and results were visualized using the DNAplotter (Carver et al., 2009) (**Supplementary Figure S1**). *S. plymuthica*, the sodorifen producer isolates and the non-producer AS9 shared high overall sequence identities, while the *S. plymuthica* PRI-2C as well as *S. proteamaculans* and *S. odorifera* were less similar (**Supplementary Table S2**). Interestingly, the non-producer *S. plymuthica* AS9 had 2901 ORFs in common with isolate 4Rx13, which was in the same range as the sodorifen producing isolates HRO-C48 and V4. Less common ORFs were found with the non-producers *S. proteamaculans* 568, *S. marcescens*, *S. plymuthica* PRI-2C, and *S. odorifera* DSM 4582. Remarkable is that although belonging to the same species, the isolate *S. plymuthica* PRI-2C differed significantly from *S. plymuthica* 4Rx13. For the search of candidate genes involved in the biosynthesis genomes of the sodorifen producers and the non-producer were analyzed. AS9 was included because of its high sequence similarity to 4Rx13 (**Supplementary Table S2**). This search identified two genes (*SOD_c13130* and *SOD_c44800*), which were analyzed via knockout mutation. The VOC profiles of these two mutants were unchanged compared to the wild type, most notably both mutants emitted sodorifen in high quantities (**Supplementary Figure S2**). Therefore this approach failed to identify candidate genes for the sodorifen biosynthesis.

### Transcriptome Analysis of *Serratia plymuthica* 4Rx13 and AS9

**FIGURE 3:**
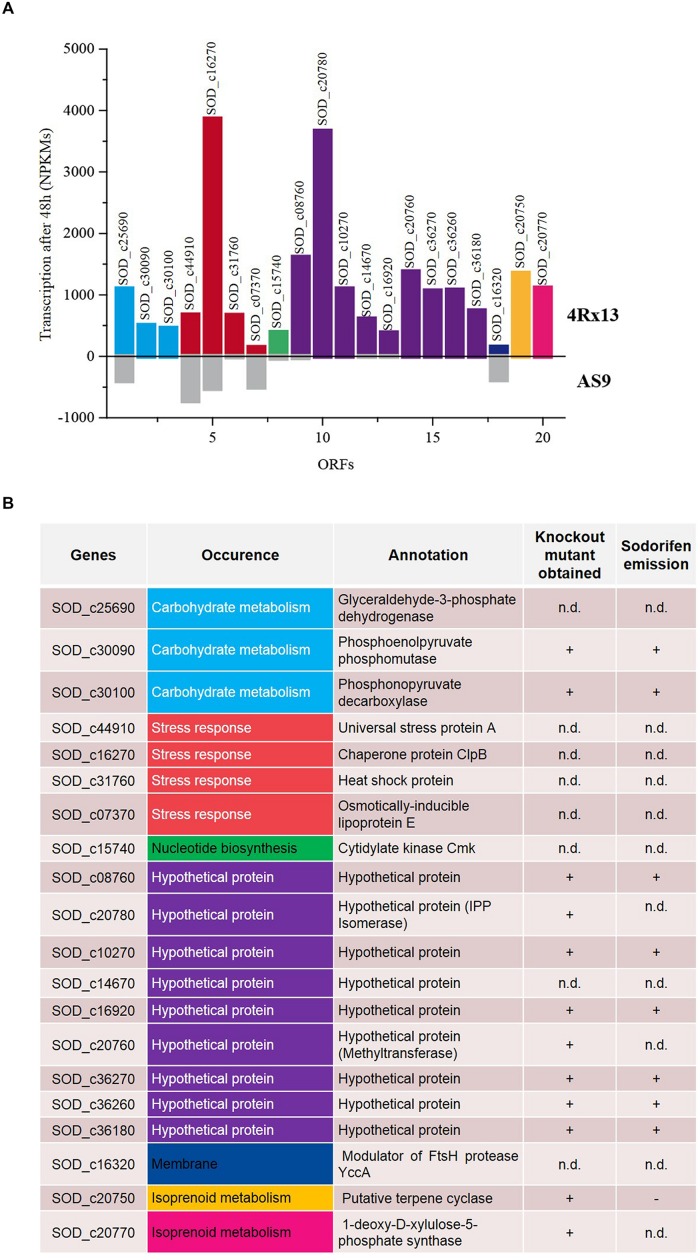
**Analysis of the transcriptomes of *Serratia plymuthica* 4Rx13 and AS9.** Both bacterial isolates were grown over 48 h in minimal medium supplemented with succinate. Samples for RNA isolation were taken after 48 h, and mRNA was sequenced to determine the expression levels of genes. **(A)** Genes expressed in high levels in *S. plymuthica* 4Rx13 (colored bars, upper panel) and expressed at low levels or not at all in *S. plymuthica* AS9 (gray bars, lower panel). Transcription activity refers to nucleotide activities per kilobase of exon model per million mapped reads (NPKM). **(B)** Differentially expressed genes in *S. plymuthica* 4Rx13 and AS9 with their annotations are listed and corresponding metabolic pathways are indicated. Summarized are the knockout mutants performed so far and their respective sodorifen emission is indicated. n.d. not determined.

**FIGURE 4:**
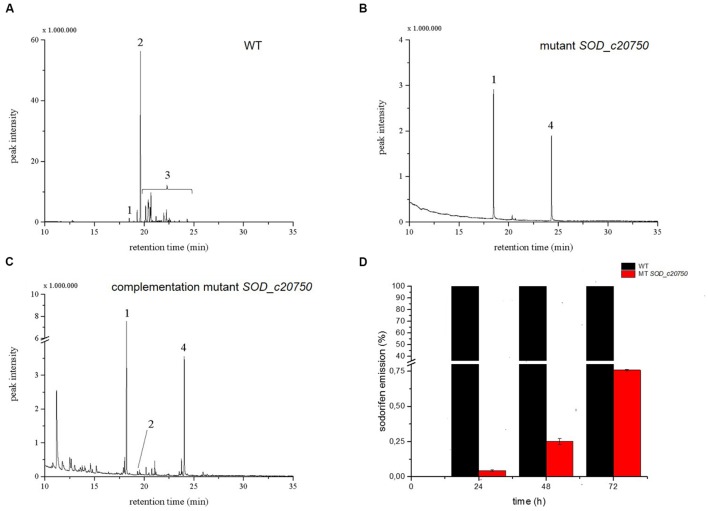
**Analysis of the *SOD_c20750* (terpene cyclase) knockout and complemented mutant of *Serratia plymuthica* 4Rx13.** Typical volatile organic compound (VOC) profiles of the headspaces of the wild type **(A)**, the *SOD_c20750* mutant **(B)** and the complemented mutant **(C)** grown in complex medium (NB) in a VOC collection system. VOCs were trapped in the time interval 24–48 h. The compounds were analyzed by GCMS and identified by comparing their mass spectra with those of the Nist107 library. (#1) nonyl acetate was internal standard (peak corresponds to 5 ng), (#2) sodorifen, (#3) sodorifen isomers, (#4) new compound. **(D)** The emission of the complemented *SOD_c20750* mutant (red) at different time points is plotted against the wild type (black), wild type equals 100%. (100% equals 274 ng of sodorifen emission or 12.21 ag sodorifen emission/cell). The peak intensity in the complemented mutant equals 0.70 ng sodorifen emission (=0.03 ag/cell). The knockout mutant did not emit sodorifen and was omitted from the graphic presentation. Error bars indicate min/max values of two clones of one complementation experiment. The emission of sodorifen was restored by introducing a recombinant plasmid consisting of the plasmid pUC19, the wild type gene *SOD_c20750* and the 500 bp upstream region of the *SOD_c20780* gene.

An alternative approach to find candidate genes involved in the sodorifen biosynthesis is the comparison of transcriptomes of the sodorifen producer *S. plymuthica* 4Rx13 and the closely related non-producer *S. plymuthica* AS9. The isolates 4Rx13 and AS9 were cultivated in a minimal medium supplemented with succinate, which was previously shown to yield very high sodorifen emissions (Weise and Piechulla unpublished). It was hypothesized that under these growth conditions genes involved in the biosynthesis of sodorifen are highly expressed allowing a distinction from housekeeping genes. Total RNA was isolated after 24 h (low sodorifen emissions) and 48 h (high sodorifen emissions) of growth of the 4Rx13 and AS9 isolates, subsequently sequenced and visualized using the TraV software tool (Dietrich et al., 2014) (**Figure 3A**). The transcription level (NPKM) of a gene to be considered as a candidate gene for sodorifen biosynthesis should be significantly higher in *S. plymuthica* 4Rx13 compared to the non-producer *S. plymuthica* AS9. In total, 20 genes were identified that met the proposed criterium. The identified genes were involved in different metabolic pathways, such as carbohydrate metabolism (three genes), stress response (four genes), nucleotide metabolism (one gene), isoprenoid metabolism (two genes), membrane modulation (one gene), and included nine hypothetical proteins (violet) (**Figure 3B**). Twelve genes have been knocked out and respective mutants were analyzed regarding their VOC profiles (**Figure 3B**). High priority was given to (i) genes of the terpenoid metabolism because many natural products are terpenes, and (ii) to hypothetical proteins because a new biosynthetic pathway was expected to be involved in the synthesis of this unsual compound. Genes of the category stress, nucleotide metabolism and membrane protein were not highly likely candidate genes/proteins and were so far not analyzed. All except one mutant exhibited the wild type phenotype and produced sodorifen (**Figures 3B** and **4A**; **Supplementary Figure S3**). The exception was gene *SOD_c20750*, a putative terpene cyclase. This gene was highly expressed in *S. plymuthica* 4Rx13 (NPKM after 48 h = 1357) but not transcribed in *S. plymuthica* AS9 (**Figure 3A**) and the knockout mutant of *SOD_c20750* emitted no sodorifen but a new compound appeared in its VOC profile (#4 in **Figure 4B**). The stability of the mutation was investigated by PCR and the presence of the resistance cassette was checked over 72 h (**Supplementary Figure S4**). The mutation of the gene *SOD_c20750* was reversed by plasmid-assisted complementation, whereby a recombinant plasmid harboring the wild type gene was reintroduced into the *S. plymuthica* 4Rx13 mutant. This gene was under the control of the native promoter (500 bp upstream region of the *SOD_c20780*, which belonged to a cluster of four genes which were further analyzed in Domik et al., 2016) and the sodorifen emission was restored (**Figure 4C**). Thus, the complemented mutant produced sodorifen, however, the quantity of sodorifen emission was significantly reduced compared to the wild type (<1% after 72 h growth) (**Figure 4D**).

### Characteristics of the Terpene Cyclase of *S. plymuthica* 4Rx13 and Homologous Genes in Other Bacteria

**FIGURE 5:**
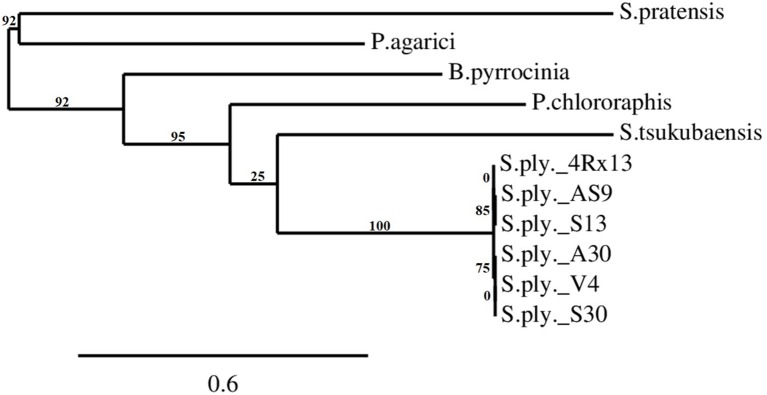
**The phylogenetic tree of *SOD_c20750* homologs (terpene cyclase) in different bacterial species and isolates.** The tree was calculated using phylogeny.fr (Dereeper et al., 2008) based on the protein sequences of the *SOD_c20750* of *Serratia plymuthica* 4Rx13. The protein sequences of the *S. plymuthica* isolates AS9, AS12, AS13, A30, V4, and S13 were aligned to the sequence of 4Rx13. *Streptomyces tsukubaensis*, *Streptomyces pratensis*, *Pseudomonas chlororaphis*, *Pseudomonas agrici*, and *Burkholderia pyrrocinia* were selected as outgroups. The tree was built on the basis of the approximation of the standard Likelihood Ratio Test (aLRT). Numbers indicate branch support values.

The *SOD_c20750* encompasses an ORF of 1154 nucleotides, which translates into a protein of 384 amino acids. The alignment and BLASTp analysis of the gene sequence with sequences stored in the NCBI and Swiss-prot databases showed that this gene exhibited high identity (99%, four amino acid differences) with other sequenced *S. plymuthica* isolates: AS9, AS12, AS13, S13, and A30 (**Table 2**). Lower identities were observed with more distantly related bacteria, such as *Pseudomonas chlororaphis* O6 (43.4%), *Streptomyces tsukubaensis* NRRL18488 (41.8%), and *Burkholderia pyrrocinia* (41%). For the latter, the proteins found in the database were annotated as hypothetical proteins. The related genes in *Pseudomonas agarici* (31%) and *Streptomyces partensis* (27.3%) were annotated as terpene synthases. A phylogenetic tree of closely related *S. plymuthica* isolates was generated online at phylogeny.fr (Dereeper et al., 2008) (**Figure 5**). A sequence with homology to *SOD_c20750* was absent in *Serratia quinivorans*, *S. proteamaculans*, and *S. odorifera* (data not shown).

**Table 2:** Homologs of *SOD_c20750* (terpene cyclase) of *Serratia plymuthica* 4Rx13 in other bacteria.

Annotation	Organism	*E* value	Identity (%) (protein)
Terpene cyclase	*S. plymuthica* 4Rx13	0.0	100
Terpene cyclase	*S. plymuthica* AS9; AS12; AS13; S13; A30	0.0	99
Uncharacterized protein	*Pseudomonas chlororaphis* O6	31e-99	43.4
Uncharacterized protein	*Streptomyces tsukubaensis* NRRL18488	190e-93	41.8
Hypothetical protein	*Burkholderia pyrrocinia*	5e-85	41
Terpene synthase	*Pseudomonas agarici*	2e-45	31
Terpene synthase	*Streptomyces pratensis*	45e-33	27.3


*The BLASTp search with the *SOD_c20750* ORF of *S. plymuthica* 4Rx13 as the query sequence was carried out with the National Center for Biotechnology Information (NCBI) Protein Database and the Swiss-prot database.*

**FIGURE 6:**
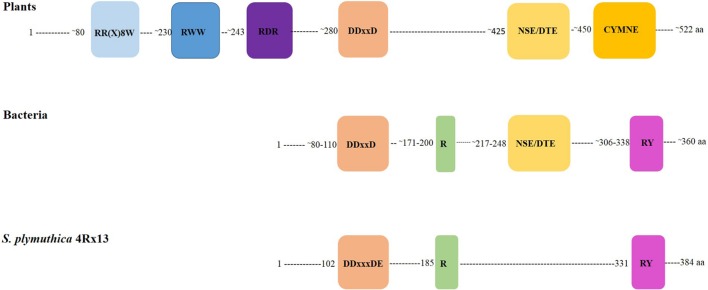
**Schematic presentation of conserved motifs in plant and bacterial terpene cyclases.** Seven conserved sequence motifs are present in plant terpene synthases. Numbers indicate the amino acids where the motif approximately appears in the protein. Plant terpene synthases are ca. 520 amino acids long. Bacterial terpene synthases are ca. 360 amino acids long and present two conserved motifs and two conserved amino acids. The *SOD_c20750* gene, annotated as terpene cyclase of *Serratia plymuthica* 4Rx13 exhibits the conserved DDXXXDE motif and the conserved R and RY amino acids of bacterial terpene cyclases.

**FIGURE 7:**
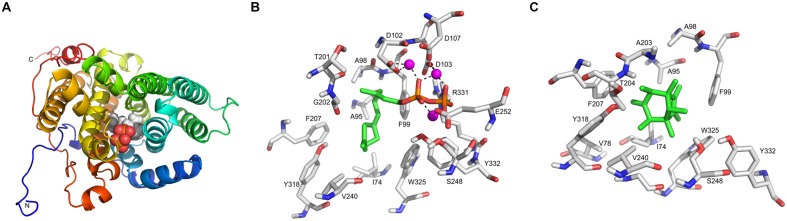
**Homology modeling of the *Serratia plymuthica* 4Rx13 terpene cyclase.** **(A)** The model of the tertiary structure of *S. plymuthica* 4Rx13 terpene cyclase is shown in rainbow representation starting from the N-terminus in blue color to the C-terminus in red color. The position of farnesyl diphosphate in the active site is highlighted in van der Waals radii representation. **(B)** Model of the active site with bound farnesyl diphosphate (green carbon atoms). **(C)** Model of the active site with bound sodorifen (green carbon atoms).

The annotation of *SOD_c20750* of *S. plymuthica* and the homologs in *P. agarici* and *S. pratensis* suggested that this gene encodes a terpene cyclase. This annotation is most likely due to the presence of the characteristic motif DDXXD, which is always found in terpene synthase sequences. Presently 63 bacterial terpene synthases are known (Dickschat, 2016), which possess four conserved motifs or amino acid residues, the aspartate-rich motif (DDXXD) (located around amino acid 80), the amino acid arginine (around 175–185), and arginine and tyrosine (ca. 310), and the NSE/DTE motif (ca. 220) (**Figure 6**). A slight variation of the aspartate-rich motif is present in the putative terpene cyclases of *S. plymuthica* 4Rx13 and AS9, which contain an additional variable amino acid X to reveal DDXXXDE. The conserved NSE/DTE motif was not found in the *SOD_c20750* gene of *S. plymuthica* 4Rx13. The model of the tertiary structure of the putative terpene cyclase of *S. plymuthica* 4Rx13 is presented (**Figure 7A**). Like the template it is an all-alpha helix fold consisting of nine almost parallel oriented helices and some rather short helices in loop regions. The position of farnesyl diphosphate (green carbon atoms) in the active site is highlighted in van der Waals radii representation (**Figure 7B**). The farnesyl moiety is recognized by several hydrophobic residues, like I74, A95, A98, F99, F207, V240, and W325. The characteristic aspartate (DDXXXD) motive consisting of D102, D103, and D107 as well as E252 binds three magnesium dications, which interact with the diphosphate moiety. Furthermore, R331 forms a salt bridge with the diphosphate. A model of the active site, harboring the product sodorifen (green carbon atoms) is shown in **Figure 7C.** The highly hydrophobic sodorifen molecule is recognized by several hydrophobic residues of the active pocket, like I74, A95, F99, F207, V240, and W325. The size and hydrophobicity of the active site strongly indicates that indeed the enzyme may catalyze this product.

Together, these structural properties of the *SOD_c20750* gene of *S. plymuthica* 4Rx13 supported that it encodes a terpene cyclase. It is the first enzyme of this class isolated from a species of Enterobacteriales. Although the biochemical characterization of *SOD_c20750* remains to be done, here it was shown for the first time that this gene is indispensable for the synthesis of sodorifen and strongly supports that sodorifen descends from the terpene metabolism. Based on the molecular formula of sodorifen (C_16_H_26_) and the proposed homology modeling it is expected that *SOD_c20750* encodes a sesquiterpene synthase rather than a monoterpene or diterpene synthase.

## Discussion

### Search for Candidate Genes Involved in the Biosynthesis of Sodorifen

It has previously been shown that the bacterium *Serratia plymuthica* 4Rx13 produced the novel compound sodorifen (Kai et al., 2010; von Reuss et al., 2010; Weise et al., 2014). However, the underlying biosynthetic pathway was still unknown. Comparative genome and transcriptome analysis of sodorifen producer and non-producer isolates generated a list of candidate genes, which might be involved in sodorifen biosynthesis. Knockout mutagenesis was applied, but many mutants continued to emit sodorifen, except the knockout of the gene *SOD_c20750*, which revealed a sodorifen-negative phenotype. For the first time a gene was identified that was involved in the biosynthesis of the unusual natural product sodorifen, which is only synthesized by three *S. plymuthica* isolates.

For the elucidation of sodorifen biosynthesis of *S. plymuthica* a multilayered approach was performed. The proteome of *S. plymuthica* 4Rx13 emitting sodorifen when grown in a complex medium was compared to the proteome under non-emission conditions (grown in NB medium supplemented with glucose), but no distinct sodorifen biosynthesis enzymes could be identified (Domik and Piechulla, unpublished results). In this publication we described a genome comparison of sodorifen producer and non-producer *S. plymuthica* isolates, but again did not find a gene involved in sodorifen biosynthesis. Only the transcriptome analysis led to successful identification of at least one gene being involved in the biosynthesis of sodorifen, the terpene cyclase gene *SOD_c20750*. As this gene is also present in the non-producer isolate AS9 it became clear why the genomic approach could not be effective for the search of candidate genes. Apparently a transcriptome analysis is a more straightforward approach, because not only silenced and repressed genes but also truncated and pseudogenes are eliminated from the pool of candidate genes. Since the transcriptome analysis indicated low transcript levels of the terpene cyclase gene we concluded that the transcription is repressed or not initiated rather than the expression of insufficient gene products. Ca. 450 nucleotides of the putative promoter regions were compared in the genomes of 4Rx13 and AS9 and several nucleotide differences or deletions were found which might be responsible for different transcription activities. To verify this hypothesis future experiments will include determination of the transcription start point and promoter exchange studies.

### Verification of *SOD_c20750* Involved in the Biosynthesis of Sodorifen

The knockout mutant of *SOD_c20750* was only successfully restored when the recombinant plasmid contained the wild type terpene cyclase gene and the native promotor of the wild type. This restoration approach has previously been used in archaea, bacteria and *Saccharomyces cerevisiae* (Shanley et al., 1986; Minet et al., 1992; Zhang et al., 2002). The complementation of the terpene cyclase mutant of *S. plymuthica* was not straight forward. In a first attempt, the wild type gene was cloned into the vector pUC19, which harbored the *lac* promoter of *E. coli.* However, the mutant remained unable to produce sodorifen. It was speculated that the transcription system of *S. plymuthica* was not able to recognize the promoter of *E. coli*, due to the different genetic background and variations of consensus sequences. Therefore, the native *gapdh* promoter was used in a second approach. However, this experiment still resulted in a sodorifen-negative phenotype. In a third attempt a promoter was selected, which was highly expressed in the transcriptome analysis. The putative promoter corresponding to the 500 bp upstream region of the gene *SOD_c20780* was selected. This approach led to a successful complementation of the mutant, resulting in the recovery of sodorifen emission. To our knowledge, this is the first successful demonstration of a complementation in *S. plymuthica*. However, the sodorifen emission was low, only less than 1% of the wild type emission level was measured. Several reasons might be responsible for the low recovery rate: (i) not much is known about plasmid gene expression in *Serratia* in general, for example the efficiency of plasmid expression might be much lower in *Serratia* compared to *E. coli*. Subsequently only low transcript levels will appear. (ii) It is very likely that other genes are involved in the biosynthesis of sodorifen, which are localized on the bacterial genome. After complementation the plasmid and genome transcription activity might not be well synchronized. (iii) The precise length of the promoter and its *cis* factors are presently not known, e.g., enhancer sequences might be missing. (iv) In the complementation construct in frame cloning was ensured and the promoter was directly fused to the reintroduced terpene cyclase gene, while ca. 3000 nucleotides are between the terpene cyclase and the promoter of SOD_c20780 in the wild type. In fact the terpene cyclase clusters together with three other genes, which are co-transcribed (Domik et al., 2016).

### Characterization of the Terpene Cyclase of *Serratia plymuthica* 4Rx13

The gene *SOD_c20750* was annotated as a terpene cyclase. While a wealth of knowledge concerning terpene cyclases of plants is available (Degenhardt et al., 2009; Chen et al., 2011), only little is known about terpene cyclases in bacteria. It is a difficult task to find bacterial terpene synthases via blast analysis, as only low levels of sequence similarities exist between bacterial terpene synthases. Yamada et al. (2015) applied a novel genome mining strategy and were able to identify 262 bacterial terpene synthases hiding in the database. Till now, 63 terpene synthases from bacteria were functionally verified (Dickschat, 2016). The majority of terpene synthases associate with microorganisms belong to Actinomycetales and only a minor fraction of Gram-negative bacteria, cyanobacteria, and myxobacteria, harbor terpene synthases (Yamada et al., 2015; Dickschat, 2016). Here we present the first terpene cyclase found in a *Serratia* species. Beside the methylisoborneol synthase of *Pseudomonas fluorescens* PfO-1 it is the second terpene synthase found in Gammaproteobacteria and the first in Enterobacteriales.

Terpene cyclases are enzymes that catalyze the final step in isoprenoid formation. They usually use geranyl pyrophosphate, farnesyl pyrophosphate, and geranyl–geranyl pyrophosphate as substrates, which are produced from the C5 precursors isopentenyl pyrophosphate and dimethylallyl pyrophosphate. These C5 building blocks are generated via the DOXP/MEP or mevalonate pathway depending which pathway is present in the bacterium (Rohdich et al., 2001). Since at least one DOXP gene (*SOD_c20770*, **Figure 3B**) was found in the genome of *S. plymuthica* 4Rx13 we assume that the precursors of the sodorifen biosynthesis result from the DOXP pathway.

Although the catalytic reaction mechanism of many plant terpene synthases are known and even crystal structures exist (Christianson, 2006; Aaron et al., 2010; Köksal et al., 2012), it is difficult to predict the correct native 3D structure of terpene cyclases or predict the product(s) just on the basis of the amino acid sequence of the enzyme (Rabe and Dickschat, 2013). According to Dickschat (2016) eight diterpene synthases and 46 sesquiterpene synthases of bacteria are presently known. The crystal structures of the pentalenene synthase, epi-isozizaene synthase, selinadiene synthase, hedycaryol synthase, and 2-methylisoborneol synthase were elucidated so far. Since the molecular formula of sodorifen is C_16_H_26_ either a sesquiterpene synthase or a diterpene synthase might be required for its synthesis, but neither the native structure or the exact mechanism of action of the terpene cyclase of *S. plymuthica* 4Rx13 is presently known, and consequently no putative biosynthesis reactions can be proposed resulting in the polymethylated bicyclic structure of sodorifen (**Figure 1**). Homology modeling was used to create a model of the tertiary structure based on a X-ray structure of a selinadiene synthase from *Streptomyces pristinaespiralis* (pdb-code 4OKZ) (**Figure 7**). Indicated by the co-crystallized dihydrofarnesyl pyrophosphate in the active site of the template the corresponding active site of the *S. plymuthica* 4Rx13 terpene cyclase could be detected (**Figure 7B**). The diphosphate moiety is recognized by three divalent magnesium cations bound to D102, D103, D107, and E252. These residues correspond to D82, D83, E87, and E232 in other bacterial enzymes (Dickschat, 2016). Furthermore, R331 is most likely involved in the diphosphate binding and superimposed with R310 in the template. Y332 may function as proton acceptor in a proton relay with the diphosphate when cleaved off to finalize the reaction cascade. The binding site of the farnesyl moiety is also highly conserved. Hydrophobic amino acid residues in the active sites of both proteins (of *S. plymuthica* and *S. pristinaespiralis*) spatially superpose very well: I74 (in 4OKZ = F55), A95 (I75), A98 (L78), F99 (F79), F207 (V187), V240 (I220), and W325 (W304). However, these differences in sequences lead to different folding and may require intermediate reaction steps of the farnesyl moiety to form the product sodorifen. In contrast, both enzymes have in common that after cleavage of the diphosphate moiety, the cation may be stabilized by the side chain of tryptophane W325 (W354), followed by a first cyclization reaction, which most likely is realized by an attack of the C1-carbon atom to the terminal dimethyl allyl group. Further reaction cascades would include other cyclization reactions and methyl shifts, which are at present highly speculative. Docking studies of the product sodorifen (**Figure 7C**) revealed the most favored docking position in the active site and clearly indicates that the structure of the active pocket may be sufficient that this compound can indeed be formed by this enzyme. There are almost perfect spatial interactions of this hydrophobic product with many of the hydrophobic amino acid side chains in the active site. These interactions and the special shape of the active site may guide and support the product formation.

A possibility to analyze the products of terpene cyclases is by heterologous expression in the genetically engineered bacterium *Streptomyces avermitilis* (Komatsu et al., 2013). It was shown that the heterologous expression of the silent biosynthetic cluster in *S. avermitilis* resulted in the production of 11 new diterpenes and two new sesquiterpenes (Yamada et al., 2015). Interestingly, *S. clavuligerus* and *S. lactacystinaeus*, which are terpene non-producers under natural conditions, showed the expression of terpene synthases in the engineered *S. avermitilis*. Thus, to unravel the function of *SOD_c20750* in the biosynthesis of sodorifen such approach has to be undertaken in the future. However, it has to be considered that (i) it is likely that not only a single gene might be sufficient to produce such a structurally unusual compound as sodorifen and (ii) the accepted substrate [isopentenyl pyrophosphate (IPP), geranyl diphosphate (GPP), methyl-GPP, neryl diphosphate (NPP), farnesyl diphosphate (FPP), and geranyl–geranyl diphosphate (GGPP), Dickschat, 2016] has to be determined. Product formation will also indicate whether the enzyme encoded by *SOD_c20750* is a single-or multi-product enzyme. Therefore, heterologous overexpression, purification, and biochemical characterization will be crucial for providing information about the function of the *S. plymuthica* terpene cyclase. The location of the active pocket and the involvement of amino acid residues that are responsible for the reaction mechanism could then be targeted using site-directed mutagenesis to investigate whether changes in single amino acids lead to novel compounds.

Bacterial terpene cyclases have no significant amino acid sequence identities to plant enzymes. Compared to typical plant terpene synthases bacterial terpene cyclases are approximately 150 amino acids shorter. Plant terpene synthases typically encompass seven conserved motifs, the RR(X)_8_W, RWW, RDR, NALV, DDXXD, NSE, and CYMNE motif (**Figure 6**, Fähnrich et al., 2014). Most prominent are the conserved DDXXD and the NSE/DTE motifs, which are involved in binding of cations (Mg^2+^), which are required for the cyclization reaction of the acyclic terpene precursor (Cane and Ikeda, 2012). The distance of ca. 140 amino acids between the DDXXD and NSE/DTE sequence is conserved in plant and bacterial terpene cyclases (**Figure 6**) (Cane and Ikeda, 2012; Yamada et al., 2015; Dickschat, 2016). Both motifs can also be found in bacterial sequences as well as conserved amino acid residues (Dickschat, 2016). The terpene cyclase of *S. plymuthica* 4Rx13 contained a slightly different DDXXXDE motif, but does not contain the NSE/DTE motif (Yamada et al., 2015; Dickschat, 2016). It is possible that the sequence (NDRYAVAMR) at position 138 is (functionally) equivalent in the isolate 4Rx13, however, this remains to be proven. Alternatively, it is also possible that the terpene cyclase of *S. plymuthica* 4Rx13 belongs to the class II enzymes, which only possess the DDXX(D/E) motif and achieve initial carbocation formation by substrate protonation rather than by substrate ionization (class I) (Christianson, 2006; Meguro et al., 2013).

Another structural feature was observed in the bacterial terpene cyclase. The comparison (**Figure 6**) showed that most of the N-terminal part of a typical plant enzymes is missing in the bacterial enzymes. While the C-terminal part of the plant terpene cyclases are responsible for the catalytic activity, the function of N-terminal part remains elusive (Christianson, 2006; Aaron et al., 2010) and therefore it is intriguing to speculate why the bacterial terpene synthase enzymes lost the N-terminal part or alternatively, why the plant enzyme gained additional sequences.

## Author Contributions

AT and RD performed the transcriptome experiment, TW established the knockout mutagenesis for *Serratia*, DD performed all other experiments, WB did the 3D modeling, DD and BP wrote the manuscript; all authors interpreted the results and approved the submitted version of the paper.

## Conflict of Interest Statement

The authors declare that the research was conducted in the absence of any commercial or financial relationships that could be construed as a potential conflict of interest.

## Footnotes

1 http://www.sanger.ac.uk/

2 https://www.chemcomp.com/
