# Supplementary material for: A Terpene Synthase Is Involved in the Synthesis of the Volatile Organic Compound Sodorifen of Serratia plymuthica 4Rx13
Source: Front Microbiol. 2016 May 19;7:737. doi: 10.3389/fmicb.2016.00737 (PMC4872519; doi:10.3389/fmicb.2016.00737)
Supplement: Supplementary file 1 [file Data_Sheet_1.PDF]

Table S1: Genome data of different *Serratia plymuthica* isolates.

Isolate	Genome size (Mb)	GC content (%)	ORFs	Reference
4Rx13	5.33	56.20	4742	Weise et al., 2014
HRO-C48	5.48	56.22	5210	Unpublished data
V4	5.51	56.20	4958	Cleto et al., 2014
AS9	5.47	56.20	5032	Tatusova et al. 2014

Table S2: Comparison of the genomes of sodorifen-producing and non-producing *Serratia* species

Serratia spp. isolate	Sodorifen producer (+) of non-producer (−)	Number of common ORFs compared to *S. ply*. 4Rx13 ORFs	Number of different ORFs compared to *S. ply*. 4Rx13 ORFs	Sequence identity (%)
S. plymuthica HRO-C48*	+	2861	556	96.5
S. plymuthica V4	+	3023	313	95.5
S. plymuthica AS9	-	2901	436	94.5
S. plymuthica PRI-2C	-	2059	1579	71
S. proteamaculans 568	-	2512	784	87.5
S. odorifera DSM 4582	-	2015	1221	74.1
S. marcescens Db11	-	2258	956	71.6

Open reading frame (ORF) sequences of *Serratia* spp. were compared using the BigBag software tool. ORFs with an e-value > −100 and a Needleman-Wunsch percent identity (NW- identity) > 70% were assigned as common ORFs, while ORFs with an e-value of e^{-20} −1 and NW identity of 0–20% were assigned as different genes. * draft genomes.

Table S3: Primer sequences and annealing temperatures

Gene	direction	T_m (°C)	Sequence (5'→3')	Primer used
SOD_c20750	sense	70	ATCAGCATACGGTCCTGCATATTGAAG<br>GCCATTGCCATTGCGGCTTCGAC<br>AATTAACCCTCACTAAAGGGCG	mutagenesis
	antisense	70	CGGTGTCCATCATCAACACGCCAGAG<br>GTGCTGGAAGGCGTGAAGCAC<br>TAATACGACTCACTATAGGGCTC	functional cassette + homology arms

SOD_c20750	sense	60	TGATCTGCTCTTTCCCCCACC	Verification of the specific gene
	antisense	61	CGTCCCAACCCGTCATGCATA	
pRED/ET	sense	55	TGAGCAGGACAATGAATCCTG	mutagenesis
	antisense	55	ACCGGTGCAAACCTCAGCAAG	
FRT-PGK-gb2-neo-FRT	sense	65	AATTAACCCTCACTAAAGGGCG	
	antisense	65	TAATACGACTCACTATAGGGCTC	
SOD_c20750	sense	50	*GAATTC ATGAACACAAATGATTTTTTGTC	complementation with *lac* promoter of pUC19
	antisense	62	*GGATCC CTAGCGCCGCCCGTACT	
Promoter + *SOD_c20750*	sense	61.9	*GAATTC GGTATTTCCTCTTACTGGCTCG	complementation with promoter overlap
	antisense	53	ATTTGTGTTCAT **GAGTGACCCCCGAAATAAAT	
SOD_c20750	sense	70	CGGGGGTCACTC ATGAACACAAATGATTT	
	antisense		*GGATCC CTAGCGCCGCCCGTACTGAT	
Promoter upstream *SOD_c20780*	antisense	68	GAGTGACCCCCGAAATAAAT	complementation
Promoter upstream *SOD_c20780*	sense	54	GGTATTTCCTCTTACTGGCTC	

* Restriction site for complementing the gene, GAATTC *Eco*RI; GGATCC *Bam*HI
** Part of overlap between gene and promotor sequence

Figure S1: The genome of *Serratia plymuthica* 4Rx13 plotted against the genomes of other *Serratia* spp. Bidirectional BiBlasts of the genomes were plotted against each other using DNAplotter (Carver et al., 2009). The circles from outside to inside are as follows: 1. open reading frames in the sense strand of *S. plymuthica* 4Rx13 (dark blue), 2. antisense strand of *S. plymuthica* 4Rx13 (light blue), 3. *S. plymuthica* HRO-C48, 4. *S. plymuthica* V4, 5. *S. plymuthica* AS9, 6. *S. proteamaculans* 568, 7. *S. odorifera* DSM 4582, 8. *S. marcescens* Db11, 9. *S. plymuthica* PRI-2C, and 10. GC content (light gray: G, dark gray: C). Red: core genome; white: pan genome. The core genome in red depicts the similarities (90–100% identity) at the nucleotide level, while white depicts the differences (0–20% identity). All sodorifen-producer isolates of *S. plymuthica* (red circles: 3-5, from outside to inside) and non-producers (circle 6-10) are shown.

Figure S2: Chromatogram of knockout mutants.
Mutants **A)** *SOD_c13130* and **B)** *SOD_c44800* were grown on NB medium in a VOC collection system and the emitted headspace volatiles were trapped in the time interval 24-48h. Volatiles were analyzed by GCMS. #1 nonyl acetate internal standard (5 ng / 10 µl), #2 sodorifen,

Figure S3: Chromatogram of knockout mutants of candidate genes for sodorifen biosynthesis on the basis of transcriptome analysis. Knockout of gene **A)** *SOD_c10270*, **B)** *SOD_c16920*, **C)** *SOD_c36260*, **D)** *SOD_c36270*, **E)** *SOD_c36180*, **F)** *SOD_c30090*, **G)** *SOD_c30100* and **H)** *SOD_c08760*. Bacteria were cultivated in a complex medium (LB). The emitted volatiles of the mutants were examined in a VOC-collection system and analyzed by GC-MS. Peaks are as follows: #1. nonyl acetate internal standard (5 ng / 10 µl), #2. sodorifen.

Figure S4: Analysis of the SOD_c20750 (terpene cyclase) knockout mutant of *Serratia plymuthica* 4Rx13. A) Polymerase chain reaction (PCR) products of the wild type gene (WT; 1.1 kb) and *SOD_c20750* mutant (MT; 2.7 kb) cultivated in complex medium over 72 h. Lanes 1–3, the mutant with the integrated resistance cassette; lanes 4–6, the wild type without the inserted resistance cassette (FRT-PGK-gb2-neo-FRT-cassette); lanes 1+4, the amplicon with gene-specific sense and antisense primers for the mutated gene; lanes 2+5, the amplicon with a specific sense primer for the inserted resistance cassette and a gene-specific antisense primer; lanes 3+6, the amplicon with a gene-specific sense primer and an antisense primer specific for the resistance cassette; M: GeneRuler 1 kb ladder. **B)** Integration of the resistance cassette (1.6 kb) was verified by PCR with specific primer combinations. The mutated gene is 1.6 kb larger (lane 2, 4, 6) than the wild type (lane 1, 3, 5) due to the integrated resistance cassette. The correct integration of the resistance cassette was verified by a combination of gene specific and resistance cassette primers. **C)** Growth curves of the mutant (dotted line) and wild type (solid line), measured as colony-forming units (CFU)/ml ($n = 2$).
