# Supplementary figures and images for: A Terpene Synthase Is Involved in the Synthesis of the Volatile Organic Compound Sodorifen of Serratia plymuthica 4Rx13
Source: Front Microbiol. 2016 May 19;7:737. doi: 10.3389/fmicb.2016.00737 (PMC4872519; doi:10.3389/fmicb.2016.00737)

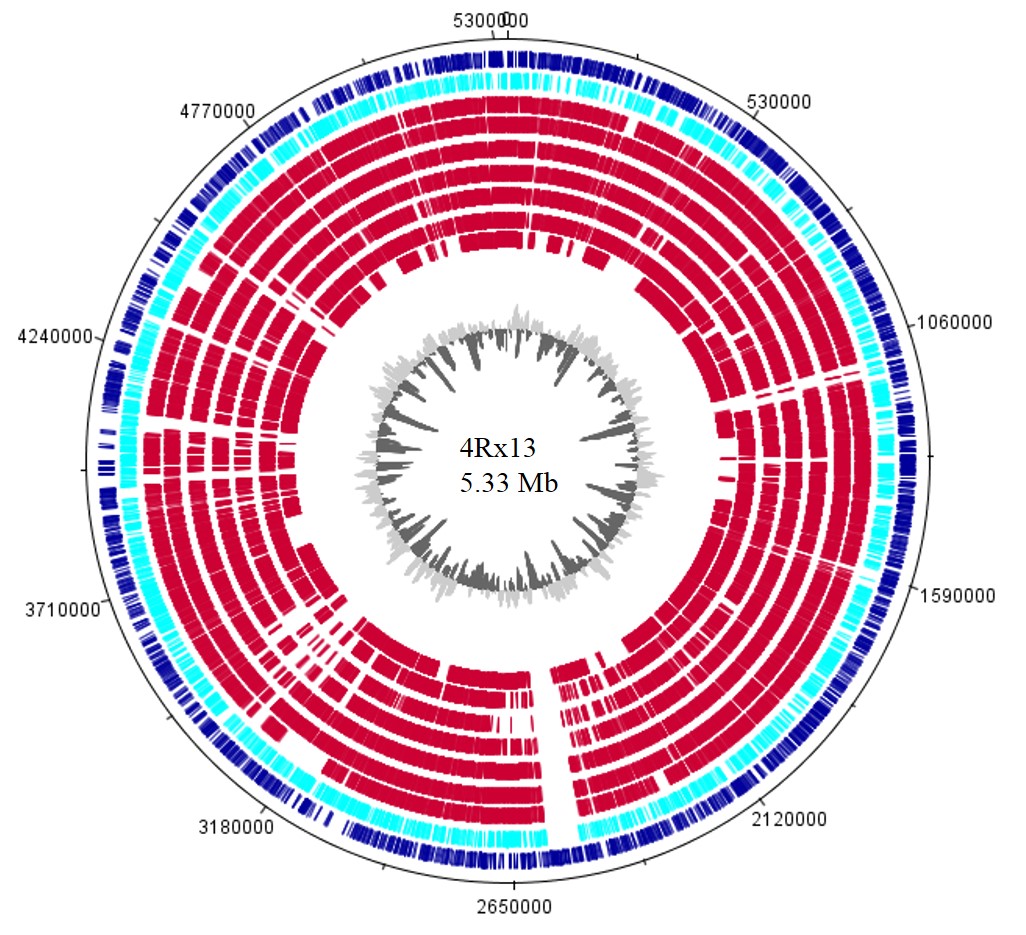

Supplement: Supplementary file 2 [file Image_1.JPEG]

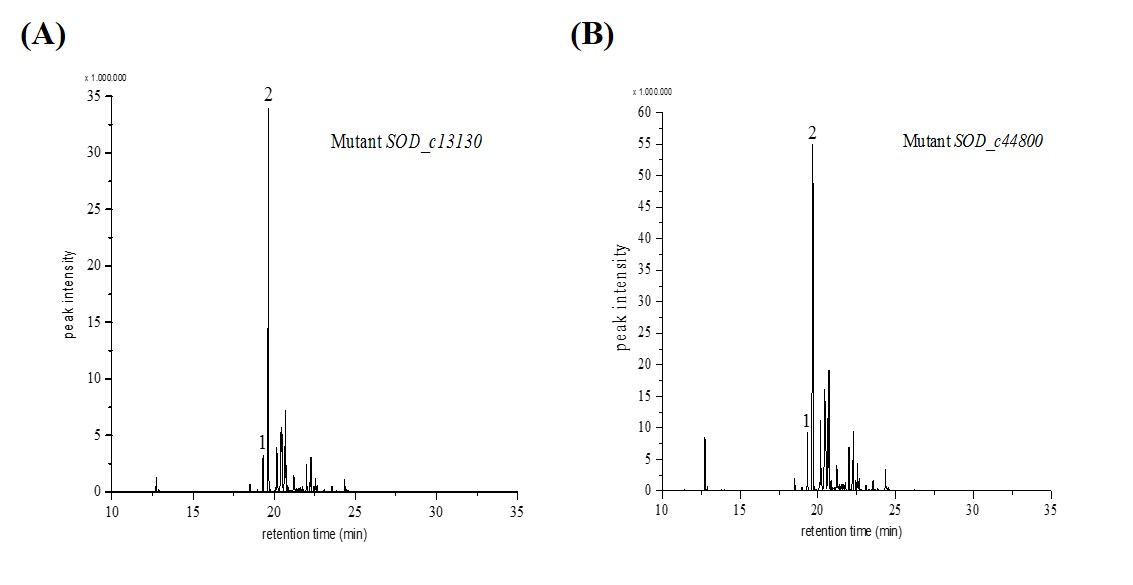

Supplement: Supplementary file 3 [file Image_2.JPEG]

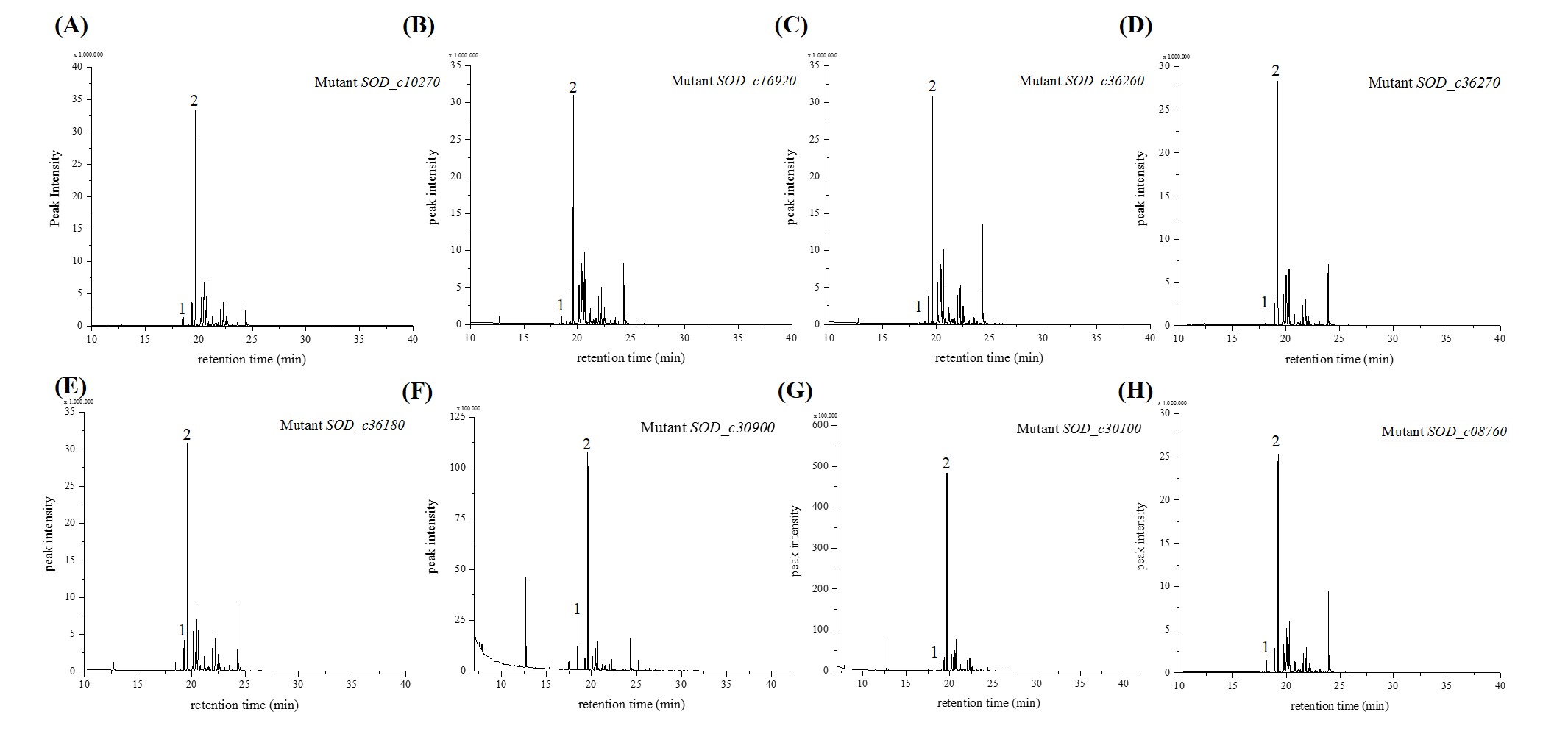

Supplement: Supplementary file 4 [file Image_3.JPEG]

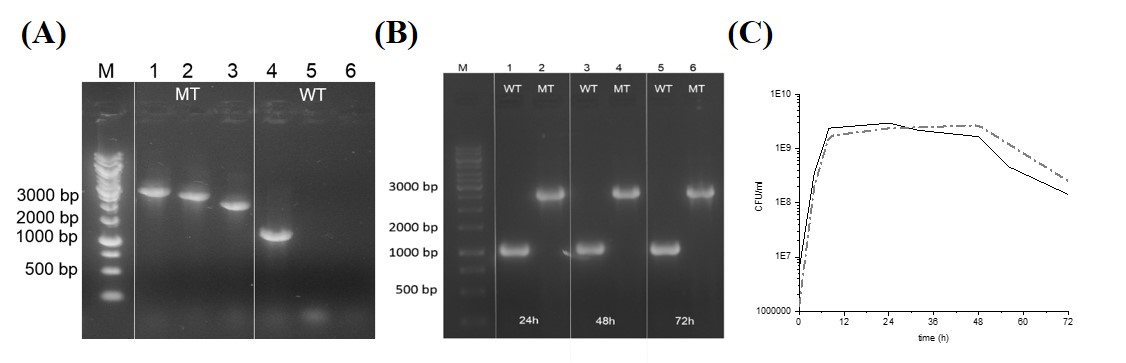

Supplement: Supplementary file 5 [file Image_4.JPEG]
